# Factors associated with childhood pneumonia and care seeking practices in Nepal: further analysis of 2019 Nepal Multiple Indicator Cluster Survey

**DOI:** 10.1186/s12889-022-14839-6

**Published:** 2023-02-07

**Authors:** Sunita Dharel, Binjwala Shrestha, Prem Basel

**Affiliations:** 1 Central Department of Public Health, Institute of Medicine, Tribhuvan University, Kathmandu, Nepal; 2 Department of Community Medicine, Maharajgunj Medical Campus, Institute of Medicine, Tribhuvan University, Kathmandu, Nepal

**Keywords:** Childhood pneumonia, Epidemiology, Healthcare seeking behavior, Nepal

## Abstract

**Background:**

Acute Respiratory Infection (ARI) is still a major public health problem in Nepal. The prevalence of ARI among under five children was 2.1% in 2019 and many children from marginalized families suffer disproportionately and many of them die without proper care and treatment. The objective of this study was to identify factors associated with childhood pneumonia and care-seeking practices in Nepal.

**Methods:**

This was a secondary analysis of the Nepal Multiple Indicator Cluster Survey (MICS) 2019, which uses multi-stage Probability Proportional to Size sampling. Data from 6658 children were analyzed using SPSS 22. Chi-square test and logistic regression analysis were conducted with odds ratio and its corresponding 95% confidence interval after adjusting for confounders.

**Results:**

Children aged 0 to 23 months had1.5 times higher odds of pneumonia compared to the age group 24 to 59 months (AOR = 1.5, CI 1.0–2.3) and children from rural area had 1.9 times the odds of having pneumonia than urban children (AOR = 1.9, CI 1.2–3.2). Underweight children had 2.3 times greater odds of having pneumonia than normal weight children (AOR = 2.3, CI 1.4–3.9). The odds of having pneumonia were 2.5 higher among children of current smoking mothers compared those with non-smoking mothers (AOR = 2.5, CI 1.1–5.7). Similarly, children from disadvantaged families had 0.6 times protective odds of pneumonia than children from non-disadvantaged families (AOR = 0.6, CI 0.4–1.0). Only one quarter of children received treatment from public facilities. Of those who received treatment, nearly half of the children received inappropriate treatment for pneumonia. One in ten children with pneumonia did not receive any kind of treatment at all.

**Conclusions:**

Pneumonia is still a public health problem in low-income countries. Public health program and treatment services should be targeted to younger children, careful attention should be given to underweight children, and awareness and nutrition related activities should be focused on rural areas. Addressing inequity in access to and utilization of treatment of childhood illnesses should be prioritized. Keywords: Childhood pneumonia, epidemiology, health care seeking behavior, Nepal.

## Background

Nepal is one of the few countries with impressive reduction of under-five mortality, with current rate of 28 deaths per 1000 live births [1, 2]. Many of these children still die from preventable causes such as diarrhea, pneumonia and other minor illnesses [3]. The latest 2019 Nepal Multiple Indicator Cluster Survey (MICS) shows that, 2.1% under-five children suffer from acute respiratory infection (ARI) [1]. The parents of many of these children do not seek timely and appropriate care, thus increasing the risk of severe illnesses and deaths. Since Nepal aims to reduce under-five mortality to 25 per 1000 live births by 2030 as part of the Sustainable Development Goals (SDGs) [4], understanding and addressing socio-economic, demographic and the underlying causes of childhood illness is important. Therefore, this study was a further analysis of Nepal MICS 2019 data to identify determinants of childhood pneumonia and care-seeking practices of their care-takers.

Studies from different countries have shown that the factors associated with childhood pneumonia are low birth weight, lack of exclusive breast feeding, crowded household environments and indoor air pollution [5–11]. Likewise, studies show that socioeconomic status, mothers’ education, exposure to household cigarette smoking, children from adolescent mothers, poor immunization, malnutrition, and area of residence accounted for a significantly higher incidence of pneumonia [6, 9, 10].

The Community-based Integrated Management of Neonatal and Childhood Illnesses (CB-IMNCI) is one of the priority public health intervention that focuses on treatment of pneumonia and management of other childhood illnesses [12].

This study identified factors associated with childhood pneumonia in Nepal and thus will provide insights to public health managers, policy makers and researchers to improve coverage of effective interventions, and thus to contribute in reduction of under-five mortality and to achieve sustainable development goals.

This study identified several variables namely; younger and underweight children, children of currently smoking mothers, mothers coming from rural but non disadvantaged areas that were not brought up in other literatures but were strongly associated with pneumonia were something considered novel. Knowing these determinants after controlling for confounding would help us to prevent pneumonia at the population level in context to Nepal as the data was of representative sample covering all ecological region of Nepal.

Considering the burden of pneumonia, associated deaths and potential to avert those deaths, critical review of causes, factors associated and care seeking needs to be identified. There were limited studies exploring these factors therefore this study attempts to contribute in generating necessary evidence on several factors associated with pneumonia and to provide recommendations for national child health programs to improve care and treatment of pneumonia and thus to contribute in child survival.

## Methods

### Study Design and setting

This study used data from Nepal MICS 2019 which is a multi-purpose cross-sectional household survey to collect internationally comparable data on the situation of children and women to monitor progress of health indicators of national development plans, the Sustainable Development Goals (SDGs) and other international commitments. Data in SPSS format (ch.sav, hh.sav, wm.sav) was downloaded from the website (https://mics.unicef.org/surveys). Primarily the data from the interview with mothers or primary caretakers of children under the age of five were utilized.

MICS covered both urban and rural areas of all seven provinces of Nepal. To create the sampling frame, MICS carried out the household listing in the enumeration area to identify households with and without children under five years and 25 households with and without under five children were selected in each sample enumeration area using systematic random sampling methods. Households with children under five were oversampled. A total sample of 512 enumeration area and 12,800 households was selected for the survey.

### Study Population

Study population for this study was under-five children. A total 6658 (unweighted sample 6749) under-five children were eligible to be included in this study, among which 139 had ARI in two weeks prior to the survey.

### Study Variables

The outcome variable used in this study was childhood pneumonia. It was categorized as “1” if presence of pneumonia i.e. an illness with a cough with rapid or difficult breathing, and whose symptoms were perceived to be due to a problem in the chest or both a problem in the chest and a blocked or runny nose in a child as reported by the mother or caretaker for a period over the two-weeks before the day of interview and “0” if no pneumonia. The independent or explanatory variables included in this study are: birth order, birth weight, age of child, sex of child, place of delivery, exclusive breastfeeding, nutritional status includes height for age, weight for age, weight for height, age of mother, maternal smoking, mother’s education, place of residence, family size, wealth index, ethnicity, media exposure, fuel use and location of the kitchen.

Age of child was categorized as 0 to 23 months and 24 to 59 months, sex was categorized as male and female, birth interval was divided into two groups (less than 2 years and more than 2 years), birth order was categorized in three groups (first or second, third or fourth and more than fourth), birth weight was categorized as less than 2500 g and equal or more than 2500 g. Likewise place of delivery categorized into two groups (institutional i.e. Hospital or health facility which includes public and private health institution, and non-institutional i.e. elsewhere than public or private health institutions), breast feeding divided into two groups (nonexclusive- if baby drink other liquid with or without mother’s breast milk and exclusive- Infants receiving breast milk, and not receiving any other fluids or foods, except oral rehydration solution, medicines, vitamins, and mineral supplements). Nutritional status includes height for age categorized as stunting and normal, weight for age categorized as underweight and normal, weight for height categorized as wasting and normal. Based on the WHO growth standard, stunting was measured based on height-for-age z-scores (≤2 standard deviation), wasting was measured based on child weight-for-height z-scores (≤2 standard deviation) and underweight was measured based on child weight-for-age z-scores (≤2 standard deviation) [13]. In mother characteristics, age of mother was classified into two category (< 20 years and ≥ 20 years) maternal smoking divided into two groups (yes-currently smokes at the time of interview and no-none smoker at that time of interview), Mother’s education also categorized into two groups (illiterate- who had no formal education and literate-either primary, secondary, or higher level of education). Similarly in household characteristics, place of residence was divided into rural and urban, Family size was categorized into two groups (up to 4 members and more than 4 members), Wealth index was categorized into 5 groups as per MICS did Household wealth status grouped into quantile: 1 = Lowest, 2 = Second 3 = Middle 4 = Fourth 5 = Highest. MICS calculate the wealth status by including Principal Component Analysis (PCA) which includes productive assets which include Hand mill, Sickle, Axe, Livestock, Hoe, Tractor, Plough, non- productive assets which include Radio, Refrigerator, TV, Bicycle, Motorbike, Phone/cell phone, Chair, Table, Bed, household utilities and other which include types of water supply, toilet, flooring, wall/house roof, light source, Person sleeping per room, Land ownership, Livestock ownership. Ethnicity was categorized into two groups (0 = “Disadvantaged” Individuals who belong to the following castes: Hill Dalit, Terai Dalit, Hill Janajati, Terai Janajati, other Terai Caste, and Muslim, 1 = “Non- disadvantaged” Individuals who belong to the following castes: Hill Brahmin, Hill Chhetri, Terai/Madhesi Brahmin/Chhetri, Newar and Other) [14, 15], Media exposure was categorized into 3 groups (1 = Poor access to media (0–3 score, based on the frequency of reading newspaper/magazine, listening radio and watching television on a daily or weekly basis) 2 = Moderate access to media (4–6 score) 3 = Good access to media (7–9 score)). In household environment related variables, fuel use divided into 2 groups (1 = Clean and safe (LPG or electric) 2 = Used solid fuel, which includes traditional solid fuel) and location of kitchen was categorized based on available of separate kitchen (0 = House with no separate kitchen 1 = House with separate kitchen in the same house or in different building).

### Method of Analysis

The downloaded data of MICS 2019 were reviewed to understand variables, variable codes, categorization and further recoding needs.

Step 1: Data from website downloaded in SPSS (*.sav) format and appropriate files (hh.sav, wm.sav, ch.sav, bh.sav) were selected.

Step 2: Appropriate variables from each file were filtered and merged into the ch.sav file. Following variables were selected from these data files:

- *hh.sav*: HH48 (family size), HC2 (ethnicity), EU1 (fuel use for cooking), EU5 (location of kitchen)
- *wm.sav*: WB4 (age of mother), MN20 (place of delivery), MN34 (birth weight), TA3 (mother’s smoking), MT1 (reading magazine), MT2 (listening radio), MT3 (watching television)
- *bh.sav*: brthord (birth order), birthint (birthinterval)
- *ch*.*sav*: CA16 (child had cough), CA17 (fast or difficulty breathing), CA18 (fast or difficulty breathing due to a problem in chest or a blocked or runny nose), CA20 (advice or treatment for pneumonia), CA21 (place or provider for pneumonia), CA23 (medicines for pneumonia), UB2 (age of child), HL4 (sex of child), BD3-BD8 (24-hr recall on feeding for 0–5 months child), melevel1 (mother’s education), HH6 (place of residence), windex5 (wealth quintile), HAZ2 (height for age), WAZ2 (weight for age), WHZ2 (weight for height)

Step 3: Dataset merged using unique identifier (HH1, HH2 in hh.sav and HH1, HH2 and LN in wm.sav and bh.sav) to ch.sav file as per MICS’s Guideline for merging data files [16].

Step 4: Variables were recoded or new variables were created as per the need of study objectives and variable definition for the study.

Step 5: Frequency distribution, chi-square analysis, bi-variate analysis, and multivariate analysis were conducted. Data were adjusted for sample weight (using chweight variable using SPSS 22) and using complex survey analysis approach (using svy command in Stata 17) during analysis, as guided by MICS methodology. Analysis was guided by the group of variables based on the literature review and findings were interpreted in the Results section.

Step 6: Data were analyzed by exclusion of missing value of following variables: family size and ethnicity (1416), media access (879), age of mothers (877), education status of mothers [2], height for age (202), weight for age [17], weight for height (181), fuel use and type of kitchen (1416). These missing values were due to collection of data from household level and nonresponse from the respondents. Birth weight and place of delivery were analyzed among the children age 0 to 23 months and exclusive breast feeding was analyzed among 0 to 5 months children.

Logistic regression analysis between dependent and independent variables were executed. Firstly, bivariate logistic regression was performed followed by multivariate logistic regression based on binary logistic regression model to adjust for the effects of other variables within the model, controlling potential confounders, and to test the strength of an association noticed in the bivariate analysis. Independent variables found to be significant in the bivariate analysis and supported by literature review were included in the multivariate analysis. Hosmer and Lemeshow goodness of fit test was carried out to ensure that the model was fit, considering the test statistic was 0.846 (*P* > 0.05).

## Results

### Descriptive Summary

As shown in Table 1, out of the total 6658 children, the majority of the children (64.9%) were from urban areas. Concerning on family size, most of the children had family members of more than four (63%). The majority were from disadvantaged ethnicity (65%). More than 72 % of the families of children had poor access to the media, i. e. regular use of radio, Television and magazine. Concerning the maternal characteristics, majority of the mothers of children (70.6%) belonged to the age group of mothers 20 to 35 years. Most of the mothers were literate (74.2%). Majority 97.2% of mothers reported that they were not current smokers. The majority were of age 24 to 59 months (61.5%), Male children were higher (52.6%) than female. In birth order, most of the children were from first birth (80.7%). More than 78% of babies had birth weight 2500 g and more than 2500 g. Nearly three-fourth (74.9%) of the children were delivered in health institution. Around two third of the children received exclusive breastfeeding. Nearly one quarter (24.5%) children were under-weight, nearly one third (31.7%) were stunted and one-in-eight (12.3%) were wasted. More than half (57.7%) of respondent households used solid fuel as main source of cooking and the rest used clean fuel. Concerning the location of the kitchen, 73.8% of the households had a separate kitchen within the same house or separate building, more than a quarter (26.2%) had no separate kitchen.

**Table 1:** Distribution and prevalence of pneumonia based on household, children’s, mother’s and household environment in Nepal

Variable	Distribution	Children with pneumonia in last two weeks				Chi square (*p*-value)
	N (%)	Yes		No		
		n	%	n	%	
**Household Related Factors**						
Place of Residence						
Urban	4318 (64.9)	68	1.6	4250	98.4	15.821 (0.00)
Rural	2340 (35.1)	71	3.0	2269	97.0	
Family Size						
≤ 4 members	1940 (37.0)	50	2.6	1890	97.4	5.596 (0.02)
> 4 members	3302 (63.0)	53	1.6	3249	98.4	
Wealth Quintile						
Lowest	1550 (23.3)	47	3.0	1503	97.0	9.466 (0.05)
Second	1366 (20.5)	24	1.8	1342	98.2	
Middle	1345 (20.2)	25	1.9	1320	98.1	
Fourth	1299 (19.5)	22	1.7	1277	98.4	
Highest	1098 (16.5)	21	1.9	1077	98.1	
Ethnicity						
Disadvantaged	3411 (65.1)	56	1.6	3355	98.4	5.882 (0.02)
Non-disadvantaged	1831 (34.9)	48	2.6	1783	97.4	
Media Access						
Poor Access	4198 (72.6)	92	2.2	4106	97.8	2.182 (0.34)
Moderate Access	1368 (23.7)	22	1.6	1346	98.4	
Good Access	213 (3.7)	3	1.4	210	98.6	
**Maternal Factors**						
Age of Mother (*n* = 5781)						
< 20 years	543 (9.4)	15	2.8	528	97.2	2.792 (0.248)
20 to 35 years	4084 (70.6)	86	2.1	3998	97.9	
More than 35 years	1154 (20.0)	18	1.5	1136	98.5	
Maternal Smoking (*n* = 6658)						
Yes	188 (2.8)	9	4.8	179	95.2	6.897 (0.01)
No	6470 (97.2)	130	2.0	6340	98.0	
Mother’s Education (*n* = 6655)						
Illiterate	1718 (25.8)	33	1.9	1685	98.1	0.322 (0.57)
Literate	4937 (74.2)	104	2.1	4833	97.9	
**Child and Birth Related Factors**						
Age (*n* = 6658)						
0–23 months	2570 (38.6)	70	2.7	2500	97.3	8.283 (0.00)
24–59 months	4088 (61.4)	69	1.7	4019	98.3	
Sex (*n* = 6658)						
Male	3502 (52.6)	82	2.3	3420	97.7	2.334 (0.13)
Female	3156 (47.4)	56	1.8	3100	98.2	
Birth order (*n* = 6558)						
1st	5293 (80.7)	104	2.0	5189	98.0	4.961 (0.08)
2nd and 3rd	1254 (19.1)	34	2.7	1220	97.3	
> 3rd	11 (0.2)	0	0.0	11	100	
Birth interval (*n* = 6558)						
*First birth*	*5305*	104	2.0	5201	98.0	4.629 (0.99)
≤ 2	785 (62.7)	18	2.3	767	97.7	
> 2	468 (37.3)	16	3.5	452	96.6	
Birth Weight (*n* = 1616)^a^						
< 2500 g	348 (21.6)	10	2.9	338	97.1	0.016 (0.97)
2500 g and more	1268 (78.4)	36	2.2	1232	97.2	
Place of delivery (*n* = 2147)^a^						
Non institutional	539 (25.1)	10	1.9	529	98.1	1.945 (0.16)
Institutional	1609 (74.9)	48	3.0	1561	97.0	
Breast Feeding (*n* = 585)^b^						
Not exclusive	221 (37.9)	8	3.6	213	96.4	5.800 (0.02)
Exclusive	363 (62.1)	3	0.8	360	99.2	
Nutritional Status						
Height for Age (*n* = 6456)						
Stunting	2045 (31.7)	53	2.6	1992	97.4	3.675 (0.05)
Normal	4411 (68.3)	82	1.9	4329	98.1	
Weight for Age (*n* = 6622)						
Underweight	1625 (24.5)	52	3.2	1573	96.8	14.571 (0.00)
Normal	4997 (75.5)	83	1.7	4914	98.3	
Weight for Height (*n* = 6477)						
Wasting	795 (12.3)	27	3.4	768	96.6	6.961 (0.01)
Normal	5682 (87.7)	111	1.9	5571	98.0	
**Household Environment Related Factors**						
Fuel Use for Cooking (*n* = 5242)						
Clean Fuel	2218 (42.3)	44	2.0	2174	98.0	0.040 (0.84)
Solid Fuel	3024 (57.7)	60	2.0	2964	98.0	
Location of Kitchen (*n* = 5242)						
No separate kitchen	1372 (26.2)	32	2.3	1340	97.7	1.150 (0.28)
Separate kitchen	3870 (73.8)	74	1.9	3796	98.1	

^a^Among 0–23 months, ^b^among 0–5 months

### Results from Regression analysis

Multivariate analysis of all factor’s significance at 95% CI in bivariate analysis was carried out using binary logistic regression. Adjusted odds ratio at 95% confidence intervals was calculated to measure the independent effects of variables.

Before adjusting potential confounder, area of residence, age of child, exclusive breastfeeding, children’s weight for age and maternal smoking were significantly associated with childhood pneumonia. Wealth status of family, ethnicity, mother’s education and fuel use for cooking also included in the multivariate analysis on the basis of published literature.

Multicollinearity test was done before multivariate analysis. In the test of multi-collinearity, none of them have tolerance < 0.1 and Variance Inflation Factor (VIF) > 10. The highest value was found to be < 10 which ensured that there was no relationship/interdependence between independent variables themselves. The condition index is also not more than 15. The goodness of fit model was assessed by Hosmer and Lemeshow test. The test statistics was 0.846 (> 0.05) that showed the model adequacy fits the data. The coefficient of determination (Nagelkerke R square) for the equation was 0.047 which means that 4.3% changes in dependent variable was due to independent variables like age of children, children with underweight, smoking habit of mothers and area of residence.

In the multivariate logistic regression model, after potential confounder were adjusted; child’s age, children’s weight for age, maternal smoking, area of residence and caste and ethnicity as the independent variable were significantly associated with childhood pneumonia at 95% CI. As shown in Table 2, children aged 0 to 23 months had 1.5 times the odds of pneumonia compared to the age group 24 to 59 months (AOR = 1.5, CI 1.0–2.3) and children from rural area had 1.9 times the odds of having pneumonia in comparison to urban children (AOR = 1.9, CI 1.2–3.2). Underweight children had 2.3 times the odds of having pneumonia than normal weight children (AOR = 2.3, CI 1.4–3.9). The odds of having pneumonia were 2.5 times higher among children of currently smoking mothers compared to those with non-smoker mothers (AOR = 2.5, CI 1.1–5.7) and children from disadvantaged families had 0.6 times protective odds of pneumonia than children from non-disadvantaged families (AOR = 0.6, CI 0.4–1.0).

**Table 2:** Adjusted and unadjusted results from multi-variate regression analysis for childhood pneumonia in Nepal

Variables	Unadjusted			Adjusted		
	OR	95% CI	*p*-value	AOR	95% CI	*p*-value
Age of child (months)						
0–23	1.6	1.2–2.2	0.00	1.5	1.0–2.3	0.03
23-59^a^						
Weight for Age						
Underweight	2.0	1.3–2.9	0.00	2.3	1.4–3.9	0.00
Normal^a^						
Maternal smoking						
Yes	2.5	1.1–5.4	0.02	2.5	1.1–5.7	0.03
No^a^						
Mothers’ education						
Illiterate	0.9	0.5–1.4	0.61	0.8	0.5–1.3	0.37
Literate^a^						
Wealth quintile						
Lowest	1.6	0.5–5.4	0.43	1.2	0.3–5.7	0.68
Second	0.9	0.3–3.3	0.92	1.0	0.2–4.8	0.97
Middle	1.0	0.3–3.3	0.98	1.0	0.3–3.8	0.95
Fourth	0.9	0.3–3.0	0.82	0.6	0.2–2.0	0.38
Highest^a^						
Place of Residence						
Rural	1.9	1.2–3.2	0.01	1.9	1.2–3.2	0.01
Urban^a^						
Ethnicity						
Disadvantaged	0.6	0.3–1.1	0.11	0.6	0.4–1.0	0.04
Non-disadvantaged^a^						
Kitchen location						
No separate kitchen	1.2	0.7–2.1	0.43	1.2	0.7–2.0	0.50
Separate kitchen^a^						
Fuel Use for Cooking						
Solid Fuel	1.0	0.5–1.9	0.95	0.6	0.2–1.3	0.16
Clean Fuel^a^						

^a^Reference analysis

Although some difference in mothers’ education, wealth quintile, caste and ethnicity, location of the kitchen and fuel use were seen none of those results were statistically significant.

### Health Care Seeking Practices

As shown in Table 3, among the children with pneumonia (*n* = 139), 12.3% of children did not seek any treatment for pneumonia. More than a quarter (26.6%) of children received treatment from a public health facility, 54.5% of children received treatment from the private health facility, 5.7% children received treatment from private pharmacy, and only a few (0.9%) children sought treatment from other sources.

**Table 3:** Care seeking practices among children with pneumonia by service providers and treatment in Nepal

Care seeking for pneumonia	Number	Percent	95% Confidence Interval
Health Care Place/Provider			
No treatment sought	17	12.3	7.4–19.8
Health Facility – Public	37	26.6	19.6–35.1
Health Facility – Private	76	54.5	44.8–63.9
Private Pharmacy	8	5.7	3.0–10.4
Other Place	1	0.9	0.1–6.2
Appropriate Treatment			
No treatment sought	14	9.8	5.5–17.0
Treated by Antibiotics	56	40.5	31.0–50.8
Not Appropriate	69	49.7	39.8–59.5

Regarding the appropriate treatment, 40.5% of children received the appropriate treatment by antibiotics. Nearly one in ten (9.8%) children with pneumonia did not receive any kind of treatment and nearly half (49.7%) of children did receive inappropriate treatment for pneumonia.

## Discussion

This study showed children from rural area had significantly higher odds of pneumonia. This finding is also supported by the findings made by other studies conducted based on the further analysis of Nepal Demographic and Health Surveys in Nepal [15, 18]. The reason behind this finding may be explained as the rural area is associated with other factors like type of home, type of fuel they use during cooking, number of family members staying at the same home, socioeconomic status and mothers’ education which in combination would affect the childhood pneumonia in rural areas.

This study showed that the childhood pneumonia was significantly associated with smoking habit of the mother. This finding was similar to the study conducted in India [19], SEAR [20] and consistent with the scientific report [21],but not consistent with the findings of study conducted in India [22]. This may be due to time spend of children with their mother and second-hand smoke exposure causes respiratory symptoms, including cough, phlegm, wheeze, and breathlessness, among under five children.

Age of child in this study was statistically significant with childhood pneumonia, both in bivariate and multivariate analysis. This finding was similar to the finding from other studies in Nepal [15], and Nigeria [23] which showed that children aged two and above were less likely to have symptoms of ARI compared to children less than age two. Similar finding was also shown in Tanzania [24] and Egypt [10]. The reason behind this finding may be due to the small airways and an immature defense system of this age group that make them more susceptible to develop pneumonia.

This study showed that the nutritional status of children was associated with childhood pneumonia. There were higher odds of pneumonia among the stunted, wasted and underweight children in comparison with normal children. Underweight was significantly associated with childhood pneumonia in both binary logistic and multivariate logistic analysis. This finding was consistent with the outcome of the study of India [19], SEAR [20], developing countries [25], Ethiopia [26] and Nigeria [23] where there were higher odds of childhood pneumonia among malnourish children. This may be due to lack of immunity among malnourished child to fight against pneumonia.

In ethnicity, increase risk of pneumonia is found significantly higher among the children from non-disadvantaged family, but the relation was not strong as enough. Literature on this is limited to support this finding.

This study showed that among the children with pneumonia, 12.3% of children did not seek any treatment for pneumonia. This finding was supported by the descriptive study conducted in Lalitpur Nepal [27]. Only a quarter (26.6%) of children received treatment from the public health facility and more than half of them received treatment from the private health facility. This finding was consistent with the finding of the community based cross sectional study conducted in India which showed that the majority of caretaker (70.5%) preferred private practitioners for the treatment of pneumonia [17]. Another cross-sectional survey conducted in Pokhara, Nepal showed that, majority sought treatment from pharmacies [28].

Regarding the appropriate treatment, 40.5% of children received an appropriate treatment by antibiotics. This study showed that nearly one in ten (9.8%) children with pneumonia did not receive any kind of treatment and nearly half of children did receive inappropriate treatment for pneumonia. This finding was not consistent with the study conducted in India which showed that the majority of the children received antibiotic for pneumonia [29] and cross-sectional survey conducted in Nepal [30] which also showed that the majority of children received appropriate treatment.

### Strength and limitation of this study

The analysis was done after accounting for the complex survey design such as sample weight. This study was based on cross-sectional data and is not intended to establish a causal relationship between the dependent and independent variables. Some questions related to the practice might not match with the real scenario it’s subject to bias to report expected behavior than a real behavior (reporting bias). Another limitation of this study is self-reporting aspect of ARI symptoms as some mother may report more symptoms than others, even they have similar symptoms at the time of interview.

## Conclusion

The survey reported that slightly more than 2% of children suffered from pneumonia in the two-weeks period preceding the survey and many of these illnesses were preventable with appropriate care and treatment.

Younger children under 2 years, underweight children, children of current smoking mothers, children from rural area and from non-disadvantaged families are more likely to suffer from pneumonia.

Caretakers of many of these children did not seek timely with appropriate care, thus increasing the vulnerability to severe illness. More children received treatment from private health facilities than public facilities.

### Recommendation

Child health programs should be targeted to provide care for younger children, and careful attention should be given to underweight and children from rural areas. Likewise, Government and supporting partners can better target their intervention and its coverage to improve care and treatment for children suffering from pneumonia.

## Data Availability

For this study data were used from publicly available data which is accessible from the MICS website (https://mics.unicef.org/surveys) on the request to UNICEF/MICS team.

## Abbreviations

ARI: Acute Respiratory Illness
CB-IMNCI: Community-based Integrated Management of Neonatal and Childhood Illnesses
CBS: Central Bureau of Statistics
CI: Confidence Interval
EA: Enumeration Area
GBD: Global Burden of Diseases
IMNCI: Integrated Management of Neonatal and Childhood Illnesses
IOM: Institute of Medicine
IRC: Institutional Review Committee
LPG: Liquified Petroleum Gas
MICS: Multiple Indicator Cluster Survey
NDHS: Nepal Demographic and Health Survey
PPS: Probability Proportional to Size
SDGs: Sustainable Development Goals
SNCU: Special Neonatal Care Unit
SPSS: Statistical Package for Social Sciences
UNICEF: United Nation’s Children Fund
WHO: World Health Organization
